# Supplementary material for: Causal Relationship between Aging and Anorexia Nervosa: A White-Matter-Microstructure-Mediated Mendelian Randomization Analysis
Source: Biomedicines. 2024 Aug 16;12(8):1874. doi: 10.3390/biomedicines12081874 (PMC11351342; doi:10.3390/biomedicines12081874)
Supplement: Supplementary file 1 [file biomedicines-12-01874-s001.zip › Supplementary Figure/Figure S4/Singe SNP plot of MR analysis for TL to ubm-b-2018.pdf]

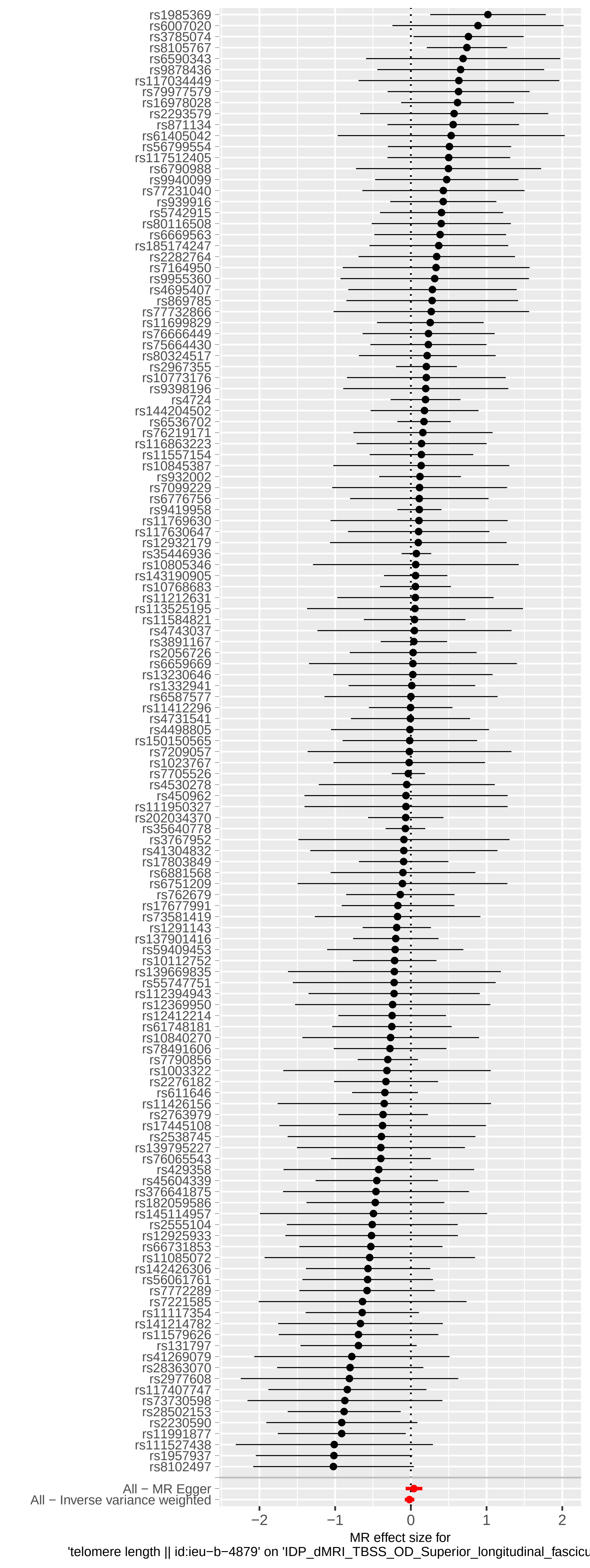

rs1985369
rs6007020
rs3785074
rs8105767
rs6590343
rs9878436
rs117034449
rs79977579
rs16978028
rs2293579
rs871134
rs61405042
rs56799554
rs117512405
rs6790988
rs9940099
rs77231040
rs939916
rs5742915
rs80116508
rs6669563
rs185174247
rs2282764
rs7164950
rs9955360
rs4695407
rs869785
rs77732866
rs11699829
rs76666449
rs75664430
rs80324517
rs2967355
rs10773176
rs9398196
rs4724
rs144204502
rs6536702
rs76219171
rs116863223
rs11557154
rs10845387
rs932002
rs7099229
rs6776756
rs9419958
rs11769630
rs117630647
rs12932179
rs35446936
rs10805346
rs143190905
rs10768683
rs11212631
rs113525195
rs11584821
rs4743037
rs3891167
rs2056726
rs6659669
rs13230646
rs1332941
rs6587577
rs11412296
rs4731541
rs4498805
rs150150565
rs7209057
rs1023767
rs7705526
rs4530278
rs450962
rs111950327
rs202034370
rs35640778
rs3767952
rs41304832
rs17803849
rs6881568
rs6751209
rs762679
rs17677991
rs73581419
rs1291143
rs137901416
rs59409453
rs10112752
rs139669835
rs55747751
rs112394943
rs12369950
rs12412214
rs61748181
rs10840270
rs78491606
rs7790856
rs1003322
rs2276182
rs611646
rs11426156
rs2763979
rs17445108
rs2538745
rs139795227
rs76065543
rs429358
rs45604339
rs376641875
rs182059586
rs145114957
rs2555104
rs12925933
rs66731853
rs11085072
rs142426306
rs56061761
rs7772289
rs7221585
rs11117354
rs141214782
rs11579626
rs131797
rs41269079
rs28363070
rs2977608
rs117407747
rs73730598
rs28502153
rs2230590
rs11991877
rs111527438
rs1957937
rs8102497
All − MR Egger
All − Inverse variance weighted
−2
−1
0
1
2
MR effect size for
'telomere length || id:ieu−b−4879' on 'IDP_dMRI_TBSS_OD_Superior_longitudinal_fascicu
